# Detection of Potentially Toxic Elements and Tolerant Native Fungi Presence in an Urban Stream in Argentina

**DOI:** 10.1111/1758-2229.70350

**Published:** 2026-05-21

**Authors:** Ludmila López Arias, Juan José Herrera, Daiana Betsabé Monzón Bechara Roucos, Nadia Stefania Alvarez, Melisa Leone

**Affiliations:** ^1^ Laboratorio de Investigación en Salud y Biodiversidad (LISyB) Universidad Nacional de Hurlingham (UNAHUR) Hurlingham Argentina

**Keywords:** bioremediation, fungal, PTEs, stream, tolerance, urban

## Abstract

Potentially toxic elements (PTEs) represent a critical threat to aquatic and terrestrial ecosystems and human health. This study assessed the presence of PTEs and the tolerance capacity of native filamentous fungi inhabiting contaminated freshwater‐associated soils. Soil and water samples were collected from four sites along the urban stream (Buenos Aires, Argentina), and PTEs concentrations were quantified. Thirteen fungal isolates were obtained from contaminated soils and characterised using phenotypic traits, while molecular identification was achieved for a subset of isolates through analyses of the internal transcribed spacer (ITS) region and the translation elongation factor 1‐alpha (TEF1‐α) gene. Probable tolerance to manganese (Mn), zinc (Zn), copper (Cu), lead (Pb), chromium (Cr) and cadmium (Cd) was evaluated on solid media using a tolerance index (TI). All isolates exhibited tolerance to at least one of the tested PTEs. Notably, three *Fusarium* strains showed tolerance to Pb, with *Fusarium equiseti*‐like IR2.1 and *Purpureocillium lavendulum*‐like MR2.8 exhibiting high tolerance across multiple PTEs. The detection of multi‐PTEs tolerance in *P. lavendulum*‐like isolates expand previous evidence largely limited to Cd and underscores the adaptive capacity of native fungal communities in contaminated urban freshwater systems, supporting their future, more detailed evaluation for potential bioremediation applications.

## Graphical abstract

Here, we report the first evidence of PTEs in the soil and surface water of the Soto stream, in the Hurlingham district of Buenos Aires, Argentina. This study provides essential baseline data for future monitoring and environmental risk assessments in the region. Our study also demonstrates the ability of native fungal communities to adapt and grow in PTEs‐contaminated environments. Notably, *Fusarium equiseti‐*like IR2.1 and *Purpureocillium‐lavenludum* like MR2.8 strains demonstrated particularly PTE tolerance, positioning them as promising candidates for future bioremediation strategies.

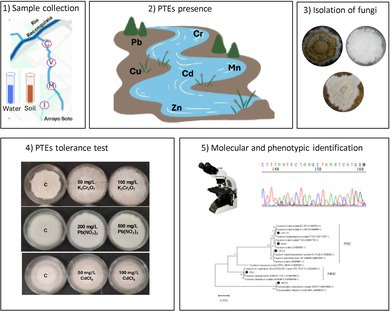

## 1 Introduction

Potentially toxic elements (PTEs) such as Cr (chromiun), Cu (copper), Cd (cadmiun), Pb (lead) and Zn (zinc) are recognised pollutants in wastewater owing to their harmful effects on environments and human health when they accumulate in soil and water systems. Common contaminants include nickel (Ni), arsenic (As), Cd, Cu, Zn, Pb and Cr, making PTEs pollution both ubiquitous and biologically hazardous (Angon et al. 2024; Hossain et al. 2025). Even at trace levels, PTEs can have significant toxicity, raising serious concerns for wastewater management and environmental protection (Mitra et al. 2022). PTEs are persistent in nature and can accumulate over time, posing risks to aquatic organisms, plants and entire ecosystems (Uchimiya et al. 2020). Their ability to bioaccumulate in living organisms, given their persistence, and, in some cases, biomagnify through the food chain further underscores the urgency of preventing their release into the environment (Mondal et al. 2018; Saidon et al. 2024).

PTEs in wastewater and soils originate from both natural processes (e.g., rock weathering, volcanic activity) and anthropogenic activities such as industrialisation, urbanisation, mining and the use of PTEs‐containing materials. Industrial and urban activities, such as metal production, vehicular emissions and improper waste disposal, are major contributors to PTEs contamination in soils and water bodies (Zaynab et al. 2022; Uchimiya et al. 2020; Rastegari Mehr et al. 2021).

In Argentina, the soil quality guideline values established by Decree 831/93 of the National Law (24.051) serve as reference thresholds for PTEs. These include Cd (3 mg/kg), Pb (375 mg/kg), Cu (150 mg/kg), Zn (600 mg/kg) and Cr (750 mg/kg). These levels are commonly used to assess environmental contamination.

Traditional physico‐chemical treatments for PTEs removal—such as precipitation, membrane filtration, adsorption and chemical oxidation–reduction are commonly applied. However, these methods are often costly, energy‐intensive and less effective at low concentrations or in large volumes of contaminated water (Qasem et al. 2021). In such cases, bioremediation can be a cost‐effective and environmentally friendly alternative. This approach exploits the metabolic activities of microorganisms, such as bacteria and fungi, to transform or immobilise PTEs (Pal et al. 2020). Fungi possess unique traits that make them well suited for PTE remediation (Khatoon et al. 2020; Vaksmaa et al. 2023). They exhibit remarkable tolerance to high PTEs concentrations and can colonise heavily contaminated soils where few organisms survive. Fungal mechanisms for PTE mitigation include bioaccumulation within their mycelium, biotransformation into less toxic or mobile forms and the secretion of extracellular enzymes that promote complexation and immobilisation (Paria et al. 2021; Noormohamadi et al. 2019). Intracellular detoxification strategies involve vacuolar sequestration, synthesis of PTEs‐binding proteins and activation of efflux systems. Furthermore, mycorrhizal fungi enhance host plant tolerance to PTEs by facilitating metal uptake and improving overall plant health (Riaz et al. 2021). Among these mechanisms, biosorption is considered the most efficient, with several fungal species—such as *Aspergillus*, *Candida*, *Trichoderma* and *Fusarium*—showing strong Cr(VI) biosorption capabilities (Al‐Asheh and Duvnjak 1995; Srivastava and Thakur 2006; Bankar et al. 2009; Elahi and Rehman 2017). Moreover, fungal‐bacterial biofilms have shown synergic effects in Cr(VI) removal, supporting their potential in integrated bioremediation strategies (Singh et al. 2016; Herath et al. 2014).

The Reconquista River basin is one of the most polluted in Argentina, with poor water quality and the presence of various contaminants well documented, including PTEs (Topalián et al. 1999; Rovedatti et al. 2001; Rigacci et al. 2013; Cantera et al. 2022). The total area of the basin is 1670 km^2^, where several watercourses converge. These tributaries traverse regions exhibiting disparities in socioeconomic characteristics and sanitation infrastructure, where urban and industrial waste may be discharged.

Urban streams in rapidly expanding metropolitan areas of South America are frequently impacted by untreated effluents and industrial activities, leading to the accumulation of PTEs in aquatic environments. These pollutants may originate from multiple anthropogenic sources, including industrial discharges, urban runoff, vehicular traffic and improper waste disposal (Cantera et al. 2022).

The aim of this study was to determine the presence of PTEs in soil and water samples from one of the Reconquista River basin's effluents and to explore the existence of native fungi with the ability to tolerate these elements.

## 2 Materials and Methods

### 2.1 Description of the Study Sites

**FIGURE 1:**
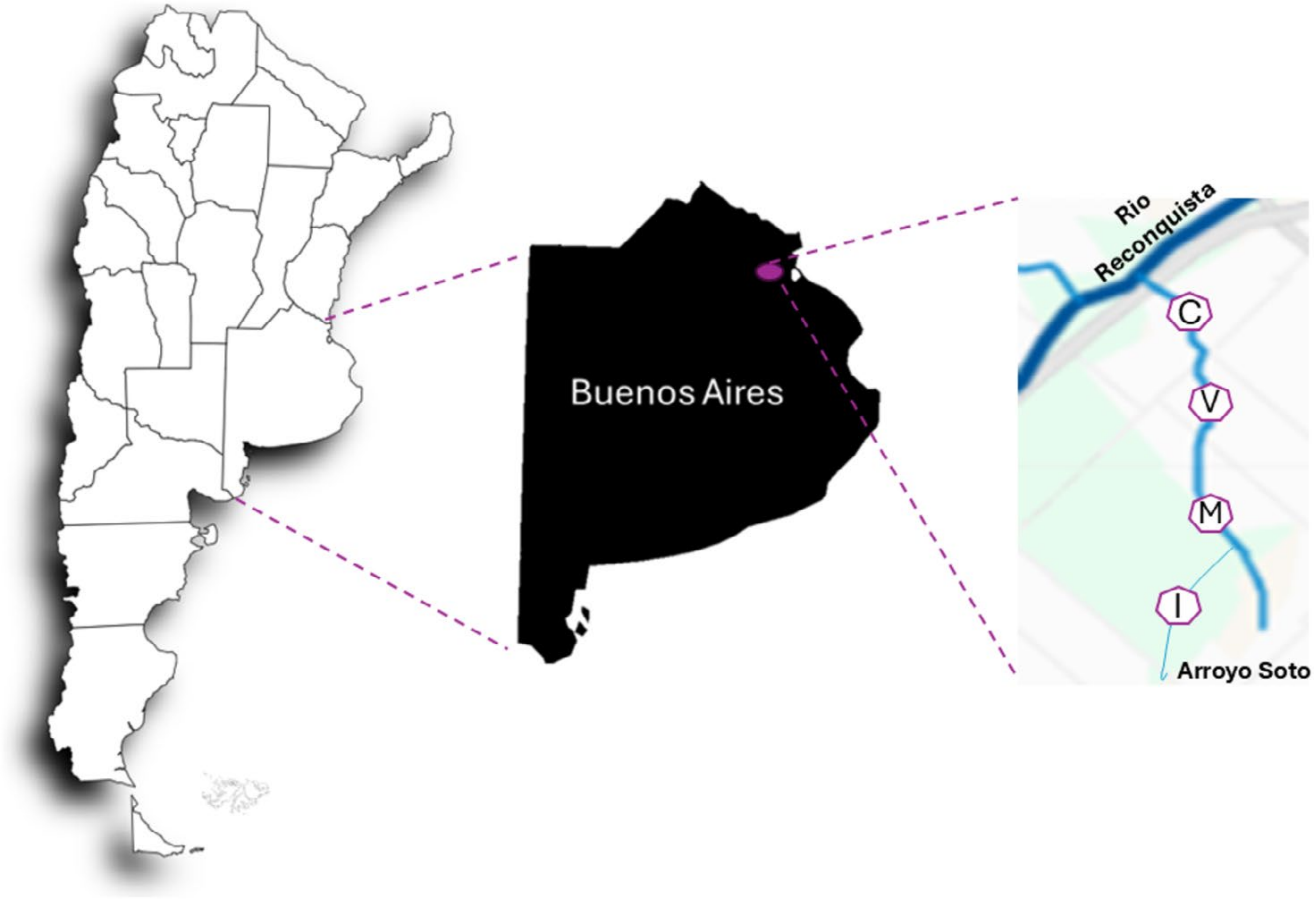
Geographic location of the sampling sites. The left panel shows the Argentine Republic. The central panel highlights Buenos Aires Province, with the purple circle indicating the western area of the Buenos Aires Metropolitan Area (AMBA). The right panel details the sampling sites (I, M, V, and C) along the Soto stream in Hurlingham district.

The Soto stream is located in the suburban area of Buenos Aires, in the east‐central region of Argentina. This watercourse is part of the middle basin of the Reconquista, and exhibits areas with varying levels of anthropogenic impact, as well as potential PTEs detection sites. The sampling sites were designated I, M, V and C. Site I (34°36′49.6″ S, 58°39′51.6″ W) is considered to have the lowest level of anthropogenic impact along the urban stream. In contrast, sites M (34°36′09.2″ S, 58°39′35.6″ W), V (34°35′08.0″ S, 58°39′43.8″ W) and C (34°34′48.1″ S, 58°39′45.2″ W) are situated in more urbanised areas, where precarious constructions have developed along the stream (Figure 1). At site V, houses are located directly adjacent to the stream bank, whereas at sites M and C, although nearby, the dwellings are somewhat more distant from the watercourse. These three sites are situated in more densely urbanised zones characterised by residential areas, road runoff and potential inputs from small industrial and commercial activities. These differences may influence the introduction and accumulation PTEs in the stream sediments.

### 2.2 Sample Collection

Surface water and soil samples were collected at I, M, V and C site using sterile plastic bottles and airtight PVC bags, respectively. At each site, one water sample from each site was collected. A 50 m transect parallel to the stream bank was established for soil sampling. Along each transect, samples were collected at four sampling points using a soil auger (10 cm depth × 6 cm diameter) and placed in clean polyethylene bags. The last samples were then combined into one composite sample. The samples were transported on ice and stored in the laboratory at 4°C in the dark until further analysis. Sampling was conducted during the winter season.

### 2.3 Analysis of PTEs in Water and Soil

Water samples (250 mL) were digested with nitric acid (HNO_3_) to prepare them for PTEs analysis. Two control samples were included in this assay: one processed identically to the water samples and another consisting of distilled water (untreated). The soil and water samples were submitted to the Servicio Geológico Minero Argentino (SEGEMAR) for analysis.

Concentrations of aluminium (Al), antimony (Sb), barium (Ba), beryllium (Be), boron (B), cobalt (Co), iron (Fe), lithium (Li), molybdenum (Mo), silver (Ag), selenium (Se), thallium (Tl), vanadium (V), As, Cd, Cu, Cr, Mn, Ni, Pb and Zn were determined using inductively coupled plasma optical emission spectrometry (ICP‐OES) following multi‐acid digestion. This assay represents an exploratory assessment based on a single sampling campaign, aimed at providing a preliminary characterisation of PTEs in this urban stream.

### 2.4 Isolation of Fungi

Fungal isolates were obtained from soil samples collected at each sampling site along the banks of the Soto stream. The isolation procedure was designed to obtain representative fungal strains from the samples for tolerance assays and potential future applications in bioremediation. Ten grams of soil were suspended in an Erlenmeyer flask containing phosphate buffered saline (PBS, 1×) and gently stirred. Serial dilutions were prepared in PBS. An aliquot of each dilution was inoculated onto Potato Glucose Agar (PGA, pH 5.6) plates. Inoculation was performed in duplicate using total extension with a Digralsky loop. Plates were incubated at 25°C for 5–7 days. Developing fungal colonies were subcultured onto fresh plates to obtain pure isolates for subsequent characterisation.

### 2.5 PTEs Tolerance Evaluation

The tolerance of fungal isolates was assessed against Cr (VI) (potassium dichromate, K_2_Cr_2_O_7_), Pb (II) (lead nitrate Pb(NO_3_)_2_), Mn (II) (manganese sulphate, MnSO_4_), Zn (II) (zinc sulphate, ZnSO_4_), Cd (II) (cadmium chloride, CdCl_2_) and Cu (II) (copper sulphate, CuSO_4_). The growth level of each isolate was classified as high growth (++), low growth (+), or no growth (−). Isolates showing growth (+ or ++) in the presence of at least one of the tested metals were selected for further analysis.

Tolerance was further evaluated by placing sterile wooden toothpicks on fungal cultures grown on PGA for 10 days. The Individual wooden sticks with adhered fungal mycelia were then placed on control plates with PGA pH 5.6 or PGA plates supplemented with sterile‐filtered metal salts at two concentrations: 200 and 500 mg/L for Pb(NO_3_)_2_, MnSO_4_, ZnSO_4_ and CuSO_4_, or 50 and 100 mg/L for K_2_Cr_2_O_7_ and CdCl_2_. Inoculated plates were incubated at 28°C for 7 days.

Fungal growth was quantified by measuring colony area (cm^2^) using ImageJ software (Rueden et al. 2017). The effect of metal on fungal growth was evaluated using the tolerance index (TI), defined as the ratio between the colony area on metal‐supplemented medium and that on control medium, calculated up to the last day of measurable mycelial growth (Ezzouhri et al. 2009; Oladipo et al. 2018). Growth in PTE‐amended media was compared with that in control plates without PTEs under identical experimental conditions. Fungal tolerance to PTEs was scored as follows: TI < 0.89 = low PTE tolerance (LPTET), TI = 0.9–1.10 = moderate PTE tolerance (MPTET) and TI > 1.10 = high PTE tolerance (HPTET). Each treatment was performed in duplicate plates (technical replicates), and the experiment was repeated three times independently to ensure reproducibility for each isolate.

### 2.6 Statistical Analysis

Analysis of variance (ANOVA) was used to compare PTEs tolerance between isolates at 5% significance level, using Infostat software (Alejandro and Rienzo 2001).

### 2.7 Microscopic and Macroscopic Characterisation

Both macroscopic and microscopic features of each fungal isolate were recorded. For microscopic observations, the adhesive tape technique was applied, which allows the visualisation fungal structures (mycelium and reproductive structures) without distortion. A strip of adhesive tape was firmly placed onto the colony surface to collect the aerial mycelium, then placed on a microscope slide with a drop of methylene blue and examine under a light microscope.

Identification at the genus level was based on a combination of macroscopic traits (colony morphology, colour, appearance and shape colony) and microscopic traits (mycelial septation, conidial shape, diameter and texture of the colony).

### 2.8 Molecular Identification

Molecular analyses were performed to complement the phenotypic characterisation and identify the isolates. Only isolates with a TI ≥ 0.9 for at least one of the PTE tested were identified using the partial sequence of internal transcribed spacer (ITS) and the elongation factor 1‐alpha (TEF‐1) gene as molecular markers.

Genomic DNA (gDNA) was extracted by harvesting mycelia into Eppendorf tubes containing 500 μL of lysis buffer (500 mM NaCl, 50 mM EDTA [pH 8.0], 100 mM Tris–HCl [pH 8.0], and 2.5% SDS). Fungal cells were mechanically disrupted using polypropylene mortars, followed by incubation at 65°C for 60 min. After incubation, 400 μL of 5 M potassium acetate was then added to each tube, and samples were incubated at −20°C for 15 min. Subsequently, 400 μL of chloroform: isoamyl alcohol (24:1) was incorporated, and the mixtures were gently inverted to homogenised. Lysates were centrifuged at 12000 *g* for 10 min to recover clear supernatants. DNA was precipitated by adding an equal volume of cold absolute isopropanol, followed by incubation at −20°C for 1 h. The samples were centrifuged at 14,000 *g* for 10 min, and pellets were washed twice with 70% cold ethanol. The resulting DNA was air‐dried at room temperature and gently suspended in 50 μL of sterile DNase‐free water.

PCR amplification of Fungal DNAs was performed in 50 μL reaction mixtures containing 1× TAS reaction buffer, 1.5 mM MgCl_2_, 200 μM dNTP mix, 1 μM of each primer (forward and reverse), 1 U Taq Holmes DNA polymerase (Inbio Highway, Buenos Aires, Argentina) and DNase‐free water. In each reaction 10–20 ng of fungal DNA was used. An ITS region was amplified using primers nu‐SSU‐0817 (5′‐TTAGCATGGAATAATRRAATAGGA), nu‐SSU‐1196 (5′‐TCTGGACCTGGTGAGTTCC) and nu‐SSU‐1536 (5′‐ATTGCAATGCYCTATCCCCA) (Borneman and Hartin 2000). The EF‐1α gene was amplified with primers EF‐1 (5′‐ATGGGTAAGGAAGACAAGAC) and EF‐2 (5′‐GGAAGTACCAGTGATCATGTT) (O'Donnell et al. 1998). PCR cycling conditions consisted of an initial denaturation at 94°C for 2 min, followed by 35 cycles of denaturation at 94°C for 30 s, annealing at 50°C–55°C for 60 s, and extension at 72°C for 1–2 min, with a final extension at 72°C for 4 min. Amplifications were performed using a Dlab TC1000 G thermal cycler (DLAB SCIENTIFIC CO. LTD).

Amplicons were sequenced by Macrogen Inc. (Seoul, South Korea) on an ABI 3730xl DNA Analyser (capillary electrophoresis). Resulting ITS and TEF‐1 sequences were manually edited using BioEdit software (version 7.7.1), and compared against the core nucleotide database from GenBank using a Basic Local Alignment Search Tool (BLASTn) (https://blast.ncbi.nlm.nih.gov/Blast.cgi).

Phylogenetic analyses were performed to assess the relationships of some isolates with reference strains from the *Fusarium solani* and *Fusarium equiseti* species complexes, *Purpureocillium lilacinum* and *Purpureocillium lavendulum* (accession numbers: FJ985408.1, KY081599.1, OR528708.1, JN235494.1, MN861794.1, LR583653.1, KY556525.1, MZ921927.1, MT465651.1, KY486704.1, KY486655.1, OQ511057.1, MH619520.1, JQ926212.1). The analyses were performed using the maximum likelihood method and Tamura‐Nei model, and branch support was estimated by bootstrap analysis with 1000 replicates. All evolutionary analyses were conducted using MEGA 11 software (Tamura et al. 2021).

## 3 Results

### 3.1 Analysis of PTEs in Water and Soil Samples

Table 1 summarises the concentrations detected of PTEs in water and soil samples collected at the different sampling sites.

**TABLE 1:** Concentrations of PTEs in water and soil samples collected from four sites (I, M, V and C) along the Soto stream.

PTE	QL	Units	Site I	Site M	Site V	Site C	QL	Units	Site I	Site M	Site V	Site C
Aluminium (Al)	1.1	μg/L	33	54	62	123	0.005	g/100 g	3	4	2.5	3.1
Antimony (Sb)	5	μg/L	< LQ				0.1	μg/g	< LQ			
Arsenic (As)	6	μg/L	< LQ				0.6	μg/g	10	6.7	1.2	5.1
Barium (Ba)	2.4	μg/L	89	86	99	127	0.24	μg/g	230	319	284	224
Beryllium (Be)	1	μg/L	< LQ				0.1	μg/g	< LQ			
Boron (B)	2	μg/L	305	322	323	303	0.2	μg/g	68	85	76	71
Cadmium (Cd)	2.4	μg/L	< LQ				0.24	μg/g	1.6	2.3	1.4	1.7
Cobalt (Co)	2.6	μg/L	< LQ				0.26	μg/g	16	21	12	11
Copper (Cu)	3.6	μg/L	< LQ				0.36	μg/g	54	86	83	54
Chromium (Cr)	1.4	μg/L	< LQ				0.14	μg/g	36	48	37	31
Iron (Fe)	1.1	μg/L	97	90	104	177	0.005	g/100 g	2.9	3.8	2.7	3.5
Lithium (Li)	9	μg/L	23	25	25	28	0.009	μg/g	26	33	22	30
Manganese (Mn)	4	μg/L	111	68	85	97	0.4	μg/g	576	769	615	475
Molybdenum (Mo)	1.2	μg/L	4	6.2	6.4	4.9	0.12	μg/g	< LQ			
Nickel (Ni)	0.9	μg/L	< LQ				0.1	μg/g	7.9	11	7.6	7.1
Silver (Ag)	5	μg/L	< LQ				0.1	μg/g	< LQ			
Lead (Pb)	7.5	μg/L	< LQ				0.75	μg/g	40	54	58	27
Selenium (Se)	9	μg/L	27	62	41	43	0.9	μg/g	< LQ			
Tallium (TI)	1	μg/L	2.9	4.3	1.2	4.3	0.1	μg/g	< LQ			
Vanadium (V)	2.5	μg/L	6	11	15	10	0.25	μg/g	93	112	84	144
Zinc (Zn)	0.73	μg/L	10	9.4	11	17	0.1	μg/g	256	345	329	158

*Note:* Water concentrations are expressed in μg/L, and soil concentrations in μg/g or g, as indicated. Light‐grey boxes represent lower concentrations and dark‐grey boxes represent the highest concentrations of each metal. Values below the limit of quantification are indicated as ‘< LQ’.

In water samples, Al and Li were detected progressively downstream (from site I to site C), with detection extending to sites near the Reconquista River. Ba, Fe and Zn were also detected at sites I, M and C. All sites revealed the presence of B, Mo, Tl, Mn, Se and V. Several elements (Cd Co, As, Cu, Cr, Ni, Sb, Be, Ag, Pb) were below the limit of quantification (LQ), though their presence cannot be excluded. Although variations among sites were observed, these differences cannot be statistically interpreted as higher or lower concentrations, since only a single (non‐independent) replicate was collected at each site.

In soil samples, no consistent spatial pattern was observed in the overall distribution of PTEs among the sampling sites. Nevertheless, several elements tended to be more concentrated at site M (Table 1). Among the analysed elements, Ba, Mn, and Zn displayed the highest overall levels. By contrast, As, Ba, Cd Co, Cu, Cr, Pb, V and Zn remained below the regulatory limits for domestic use. Mo, Tl, Sb, Be, Ag and Se were below the LQ.

### 3.2 Qualitative Screening

**FIGURE 2:**
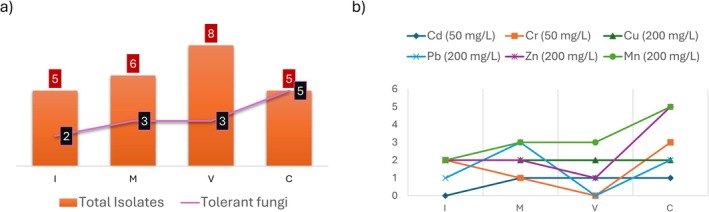
(a) Total number of fungal isolates (orange bars) and number of Potentially Toxic Elements‐tolerant isolates (purple line) obtained from each sampling site (I, M, V and C) along the Soto stream. (b) Number of isolates per site showing tolerance to each tested metal: Cd and Cr: 50 mg/L; Cu, Pb, Zn and Mn: 200 mg/L.

Fungal strains isolated from soil at Sites I, M, V and C were screened for tolerance (visible growth) to Cr (VI), Pb (II), Zn (II), Cd (II), Mn (II) and Cu (II). Initial qualitative screening identified 24 tolerant strains (Table 2)—five from Site I, six from M, eight from V and five from C—that grew (+, ++) on agar plates supplemented with at least one PTE (Figure 2a). Of these fungal strains, 13 exhibited high growth (++) in the presence of at least one of the tested elements.

**TABLE 2:** PTEs tolerance of fungal isolates exposed to cadmium (Cd), chromium (Cr), copper (Cu), lead (Pb), zinc (Zn) and manganese (Mn) at the indicated concentrations.

Isolation	Cd (50 mg/L)	Cr (50 mg/L)	Cu (200 mg/L)	Pb (200 mg/L)	Zn (200 mg/L)	Mn (200 mg/L)
IR1.13	+	+	+	+	+	ND
**IR2.1**	+	**++**	**++**	+	**++**	**++**
IR2.3	+	—	+	+	+	ND
**IR2.5**	+	**++**	**++**	**++**	**++**	**++**
IR2.8	+	+	+	+	+	ND
MF	+	+	+	+	+	ND
**MC**	+	+	**++**	**++**	+	**++**
**MR1.4**	+	—	+	**++**	**++**	**++**
MR2.4	+	+	+	+	+	ND
MR2.6	+	+	+	+	+	ND
**MR2.8**	**++**	**++**	**++**	**++**	**++**	**++**
**VA**	+	+	+	+	+	**++**
VB	+	+	+	+	+	ND
VR1.1	+	+	+	+	+	ND
VR1.2	+	+	+	+	+	ND
**VR1.10**	+	+	**++**	+	+	**++**
VR2.1	+	+	+	+	+	ND
VR2.2	+	+	+	+	+	ND
**VR2.8**	**++**	+	**++**	+	**++**	**++**
**CD**	+	+	**++**	+	**++**	**++**
**CR1.3**	+	+	+	+	**++**	**++**
**CR1.4**	+	**++**	+	+	**++**	**++**
**CR1.13**	**++**	**++**	+	**++**	**++**	**++**
**CR2.2**	+	**++**	**++**	**++**	**++**	**++**

*Note:* Symbols denote growth performance: ‘+’ = low growth, ‘++’ = high growth and ‘−’ = no growth in the presence of each PTE. Growth was visually assessed after incubation on PGA supplemented with the respective metal. “ND” (no data) indicates that the isolate was not evaluated for that PTE. Grey boxes indicate isolates that showed high tolerance in the presence of each specific PTE. Bold values indicate the isolates selected for evaluating the tolerance index against differretn PTEs.

PTEs‐specific patterns were observed. Remarkably, at Site C, every isolate showed growth in the presence of at least one PTE (Figure 2a). No isolates from Site I tolerated Cd (Figure 2b and Table S1). Similarly, none of the isolates from Site V tolerated Cr or Pb under the evaluated concentrations. In the presence of Cr, two isolates from Site I, one from Site M, and three from Site C tolerated this metal. Cu tolerance was evident for two filamentous fungal isolates from each site. Pb tolerance was detected in one isolate from Site I, three from Site M and two from Site C (all showing high growth), but not in any isolate from Site V. Regarding Zn, two isolates grew at both Sites I and M, only one at Site V and all isolates from Site C displayed high growth. In the presence of Mn, two isolates from Site I and three each from Sites M and V showed high growth, while all isolates from Site C exhibited robust tolerance (Figure 2b).

### 3.3 PTEs Tolerance Test

**FIGURE 3:**
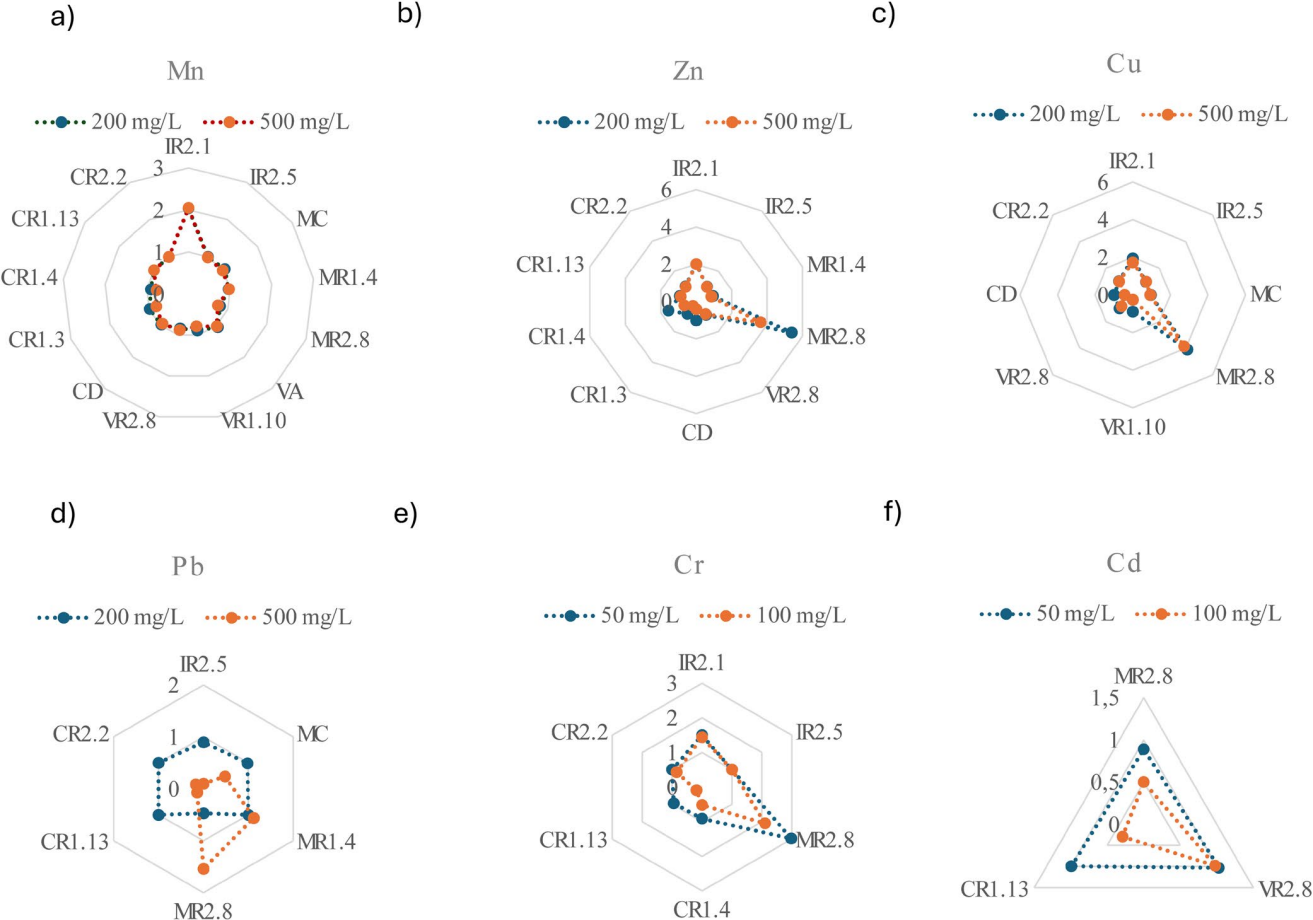
Radar charts showing the tolerance index (TI) of selected fungal isolates in the presence of increasing concentrations of PTEs. Growth was assessed on PGA plates supplemented with: (a) Mn, 200 and 500 mg/L; (b) Zn, 200 and 500 mg/L; (c) Cu, 200 and 500 mg/L; (d) Pb, 200 and 500 mg/L; (e) Cr, 50 and 100 mg/L and (f) Cd, 50 and 100 mg/L. Dashed blue lines indicate growth at the lowest concentration tested, while dashed orange lines represent growth at the highest concentration. Numeric values represent the TI under each condition.

To calculate the tolerance index (TI), we monitored the growth of the 13 isolates that showed high growth (++) over successive days. TI values for Mn, Zn, Cu, Pb, Cr and Cd are shown in Figure 3 and detailed Table S2.

In the presence of Mn, most isolates maintained a TI close to 1 at 200 mg/L and 500 mg/L, except isolate IR2.1, which showed high PTE tolerance (HPTET; TI ≅ 2; Figure 3a). Regarding Zn, isolates IR2.1, MR2.8 and CR1.4 exhibited HPTET (TI ≥ 1.57) at 200 mg/L, whereas CR1.3, CR1.4 and VR2.8 displayed notably lower TIs at 500 mg/L (Figure 3b). Cu tolerance was generally moderate and, in some cases, low (VR1.10, VR2.8 and CD), with most isolates maintain TI values close to 1 at both concentrations. However, IR2.1 and MR2.8 showed HPTET (TI ≥ 1.73; Figure 3c). Pb tolerance was more variable: MR1.4 and MR2.8 showed HPTET at 500 mg/L, while CR1.13 and CR2.2 showed low tolerance (LPTET: TIs < 0.89; Figure 3d). For Cr, most isolates exhibited moderate PTE tolerance (MPTET) at the lowest evaluated concentration (50 mg/L), but decreased growth at 100 mg/L; IR2.1 and MR2.8 again stood out as the most tolerant strains (Figure 3e). In the case of Cd, only three isolates were tested. VR2.8 maintained MPTET at both 50 and 100 mg/L, whereas MR2.8 exhibited LPTET (TIs < 0.9), indicating Cd sensitivity at both concentrations (Figure 3f).

**FIGURE 4:**
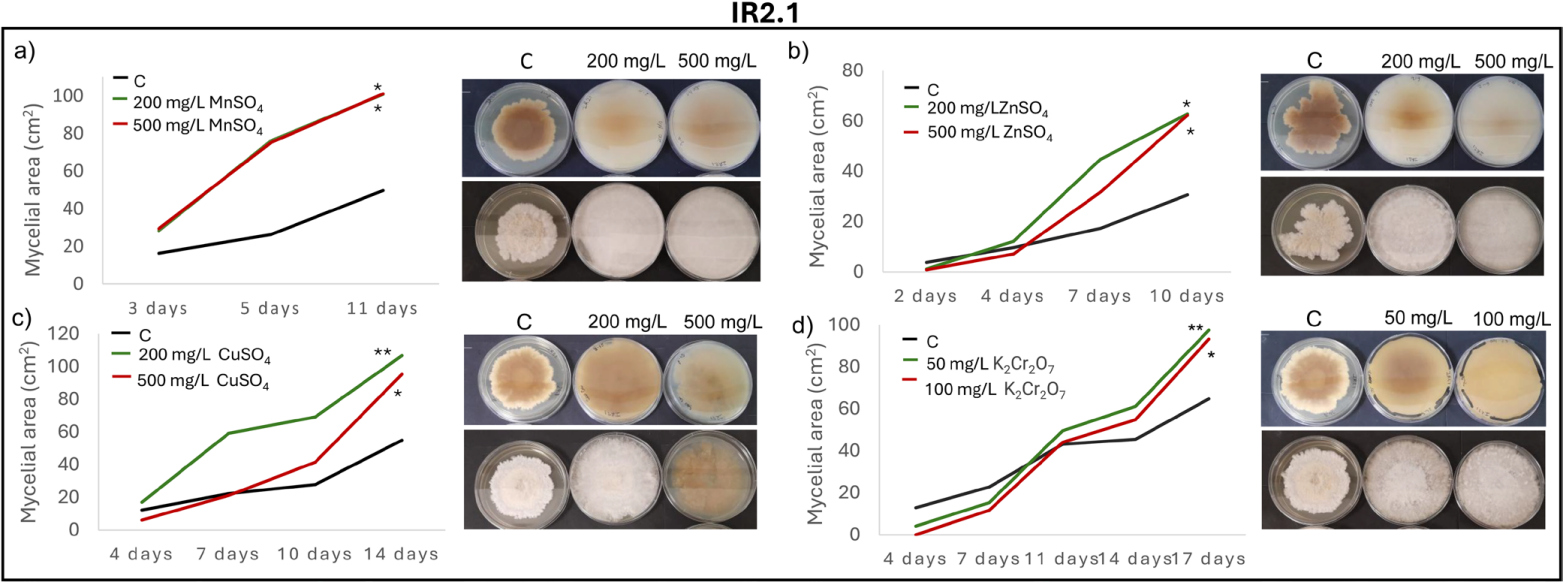
Mycelial growth kinetics of IR2.1 in the presence of different PTEs at two concentrations. (a) Mn, (b) Zn, (c) Cu and (d) Cr. Line graphs depict the increase in mycelial area (cm^2^) over time on PGA control medium (C) and PGA supplemented with the corresponding PTE. Asterisks (*/**) indicate significant differences compared with the control (*p* < 0.05). Representative images illustrate colony morphology under control and PTE stress conditions. Scale bars (upper left corner) represent 1 cm.

**FIGURE 5:**
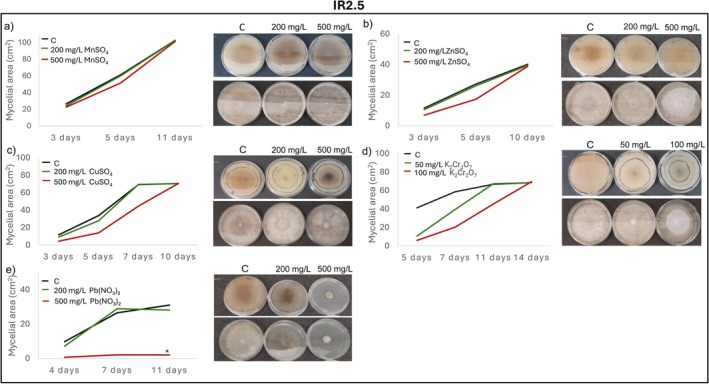
Mycelial growth kinetics of IR2.5 in the presence of different PTEs at two concentrations. (a) Mn, (b) Zn, (c) Cu, (d) Cr and (e) Pb. Line graphs depict the increase in mycelial area (cm^2^) over time on PGA control medium (C) and PGA supplemented with the corresponding PTE. Asterisks (*) indicate significant differences compared with the control (*p* < 0.05). Representative images illustrate colony morphology under control and PTE stress conditions. Scale bars (upper left corner) represent 1 cm.

**FIGURE 6:**
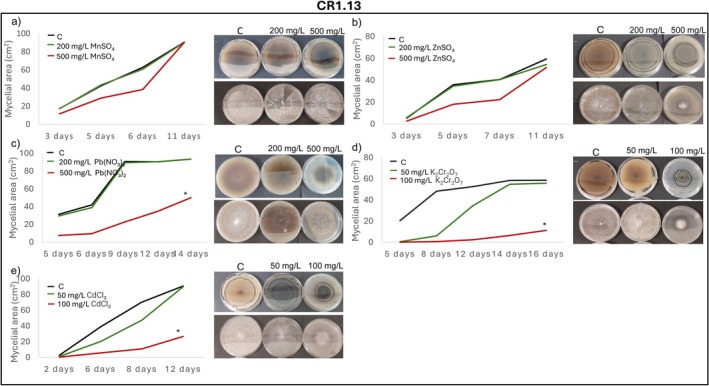
Mycelial growth kinetics of CR1.13 in the presence of different PTEs at two concentrations. (a) Mn, (b) Zn, (c) Pb, (d) Cr and (e) Cd. Line graphs depict the increase in mycelial area (cm^2^) over time on PGA control medium (C) and PGA supplemented with the corresponding PTE. Asterisks (*) indicate significant differences compared with the control (*p* < 0.05). Representative images illustrate colony morphology under control and PTE stress conditions. Scale bars (upper left corner) represent 1 cm.

**FIGURE 7:**
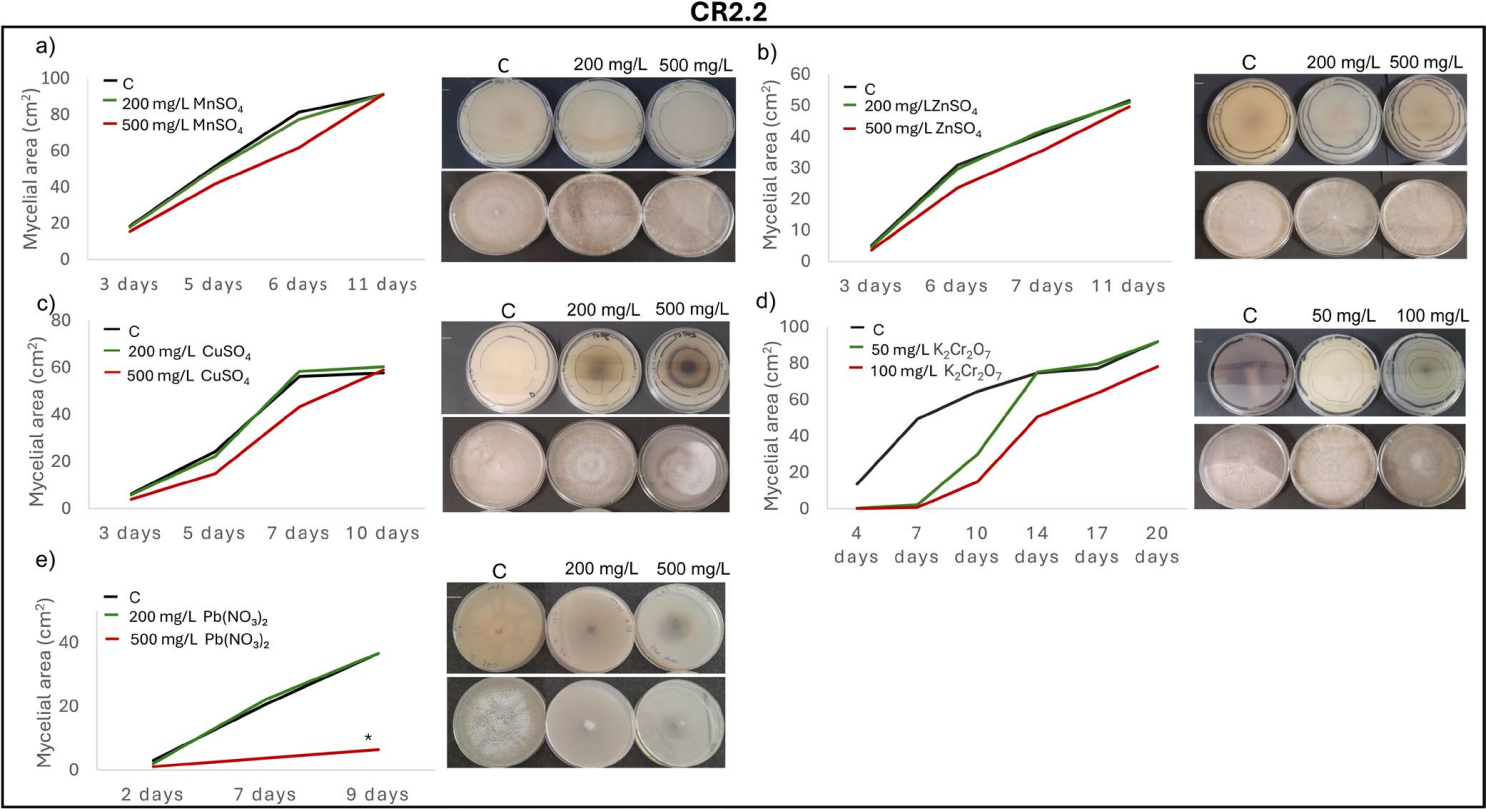
Mycelial growth kinetics of CR2.2 in the presence of different PTEs at two concentrations. (a) Mn, (b) Zn, (c) Cu, (d) Cr and (e) Pb. Line graphs depict the increase in mycelial area (cm^2^) over time on PGA control medium (C) and PGA supplemented with the corresponding PTE. Asterisks (*) indicate significant differences compared with the control (*p* < 0.05). Representative images illustrate colony morphology under control and PTE stress conditions. Scale bars (upper left cover) represent 1 cm.

**FIGURE 8:**
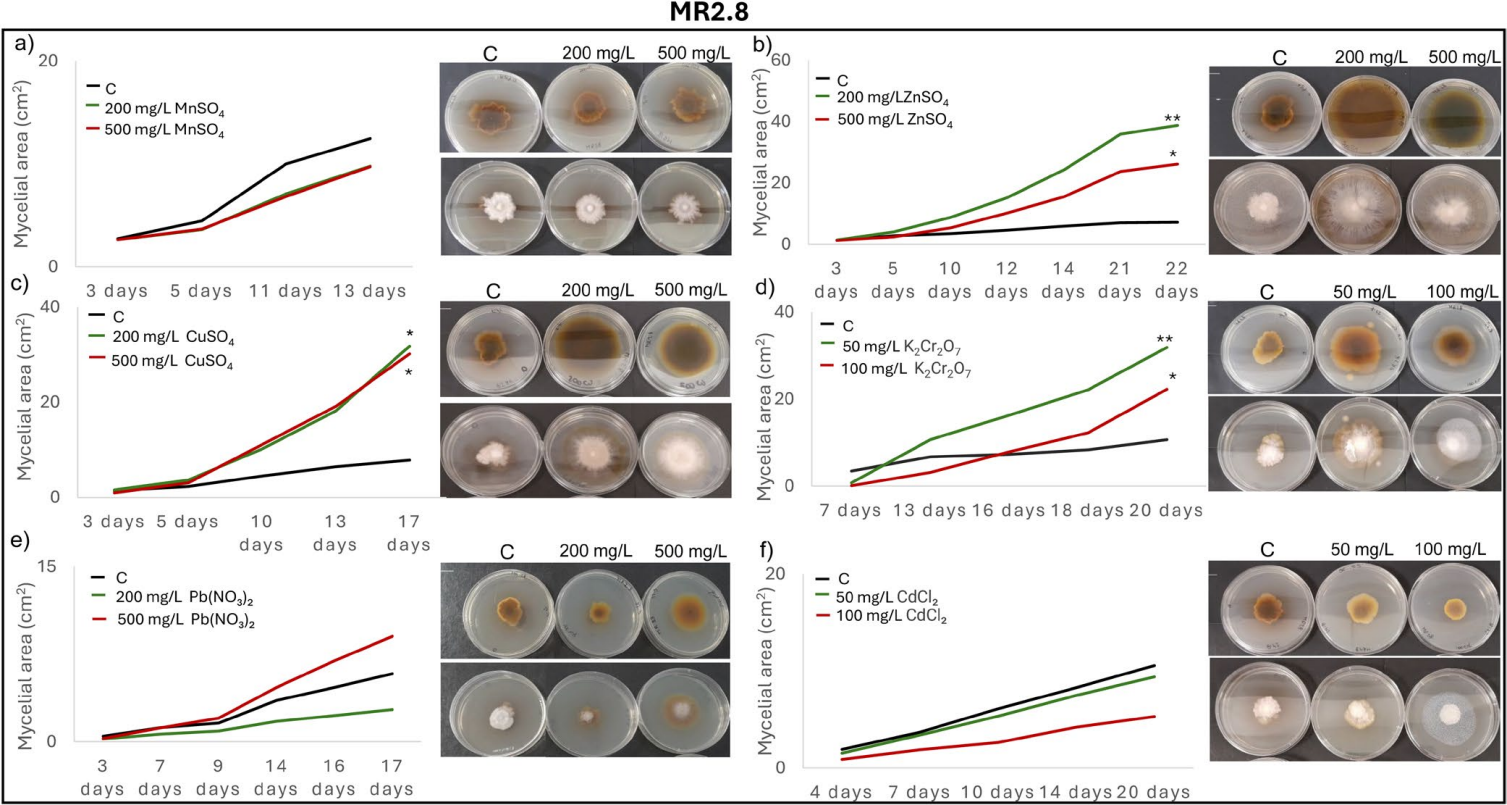
Mycelial growth kinetics of MR2.8 in the presence of different PTEs at two concentrations. (a) Mn, (b) Zn, (c) Cu, (d) Cr, (e) Pb and (f) Cd. Line graphs depict the progression of mycelial area (cm^2^) over time on PGA control medium (C) and PGA supplemented with the corresponding PTE. Asterisks (*/**) indicate significant differences compared with the control (*p* < 0.05). Representative images illustrate colony morphology under control and PTE stress conditions. Scale bars (upper left corner) represent 1 cm.

Radial mycelial growth assays further confirmed isolate‐specific tolerance patterns. IR2.1 showed significantly enhanced growth relative to the control in the presence of Mn, Zn, Cu, and Cr after 11, 10, 14 and 17 days of incubation, respectively (Figure 4). IR2.5 displayed growth comparable to the control in the presence of Mn, Zn, Cu, Cr and Pb, except for a marked inhibition at 500 mg/L of Pb (Figure 5). CR1.13 maintained control‐like growth in the presence of Mn and Zn and at the lowest concentrations of Pb, Cd and Cr, but exhibited reduced growth at higher levels of Pb, Cd and Cr (Figure 6). CR2.2 showed no mycelial inhibition at both concentrations of Mn, Zn and Cu, or at low Cr and Pb, but showed increasing sensitivity at higher Cr and Pb concentrations, with a particularly pronounced sensitivity at high Pb levels (Figure 7). MR2.8 exhibited heterogeneous responses: enhanced mycelial growth in the presence of Zn, Cu, and Cr; control‐like growth with Mn and Cd; and reduced growth at low Pb and, surprisingly, greater growth than the control at 500 mg/L of Pb (Figure 8). The MC isolate tolerated low concentrations of Mn, Cu and Pb, but was highly sensitive at 500 mg/L (Figure S1). MR1.4 showed a similar trend, although in this case, Zn rather than Cu supported growth comparable to the control (Figure S2).

VR2.8 exhibited control‐like growth at both concentrations of Cd, but slightly reduced growth with Mn Zn and higher concentrations of Cu (Figure S3). CR1.4 tolerated Mn and low Cr but was inhibited at higher Cr. With Zn, its mycelial growth was markedly enhanced at low concentrations, but its radial growth was inhibited at higher levels (Figure S4). CR1.3 and CD isolates exhibited control‐like mycelial growth at low concentrations of Zn and Cu, while VR1.10 displayed similar growth at low Mn (data not shown).

### 3.4 Molecular and Phenotypic Identification of Fungi Tolerant to PTEs

Fungal isolates showing MPTET or HPTET to at least one PTE were identified based on molecular and phenotypic studies. The most diverse group recovered as culturable and metal‐resistant fungi from the investigated sites belonged to the phylum Ascomycota.

**FIGURE 9:**
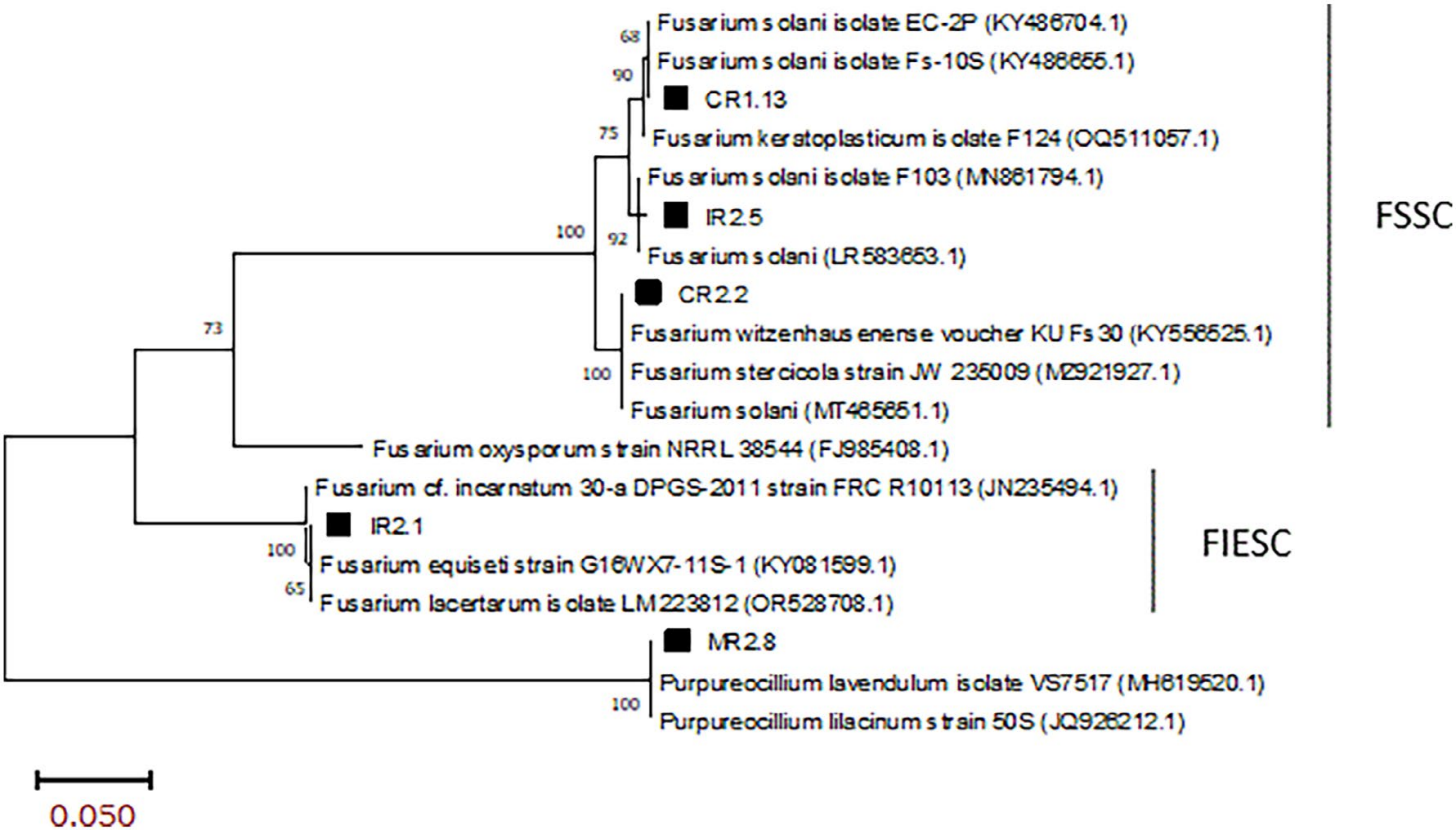
Phylogenetic tree based on the TEF‐1 gene from *Fusarium* and *Purpureocillium* species. The evolutionary history was inferred using the maximum likelihood method and the Tamura‐Nei model. The percentage of trees in which the associated taxa clustered together is shown next to the branches. Branch values below 50 are not shown.

**FIGURE 10:**
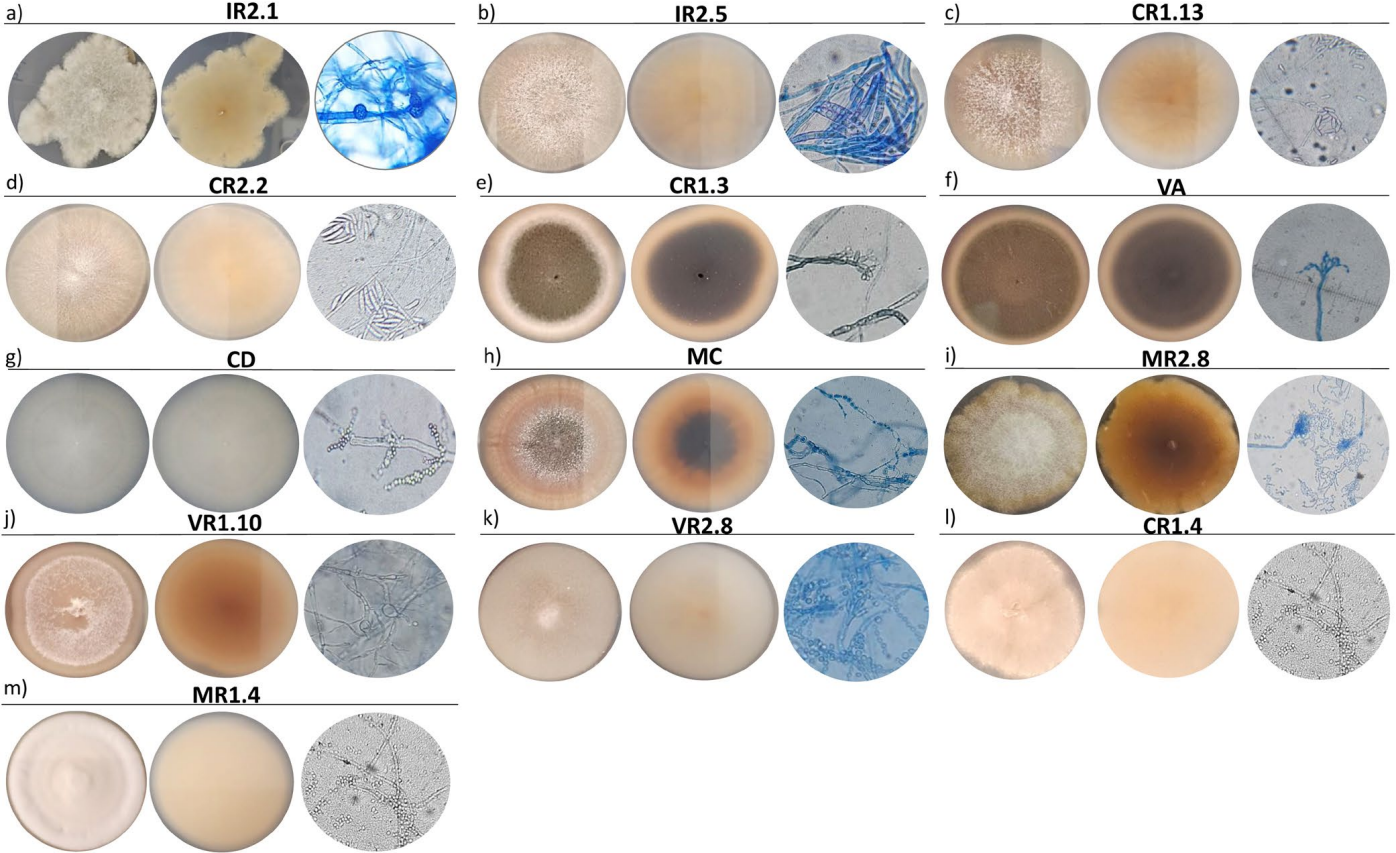
Macroscopic and microscopic characterisation of the 13 native fungal isolates. For each isolate, three panels are shown (from left to right): Colony morphology on PGA, reverse side of the colon, and microscopic features. (a) IR2.1, (b) IR2.5, (c) CR1.13, (d) CR2.2, (e) CR1.3, (f) VA, (g) CD, (h) MC, (i) MR2.8, (j) VR1.10, (k) VR2.8, (l) CR1.4 and (m) MR1.4.

Macroscopic and microscopic studies, in conjunction with molecular analyses, revealed that four of the 13 isolates belonged to the genus *Fusarium*. Phylogenetic analysis based on the EF‐1α gene showed that isolates CR1.13, IR2.5 and CR2.2 clustered within the *Fusarium solani* species complex (FSSC), while IR2.1 clustered with the *Fusarium incarnatum‐equiseti* species complex (FIESC). Specifically, IR2.1 showed 100% identity with three species: *Fusarium incarnatum*, *Fusarium equiseti* and *Fusarium lacertarum* (Figure 9). This isolate produced an irregular, white, cottony colony with a slightly raised centre and a cream‐coloured back, whose microscopic examination revealed the presence of macroconidia (Figure 10a).

Within the FSSC group, CR1.13 and IR2.5 clustered with *Fusarium solani* isolates, exhibiting 68% and 92% identity, respectively (Figure 9). CR2.2 showed 100% identity with three FSSC members: *Fusarium witzenhausenense*, *Fusarium stercicola* and *Fusarium solani*. In addition, according to the analysis with TEF‐1 primers, MR2.8 belonged to the *Purpureocillium* genus (Figure 9). The nucleotide sequences of the different obtained isolates have been submitted to GenBank (accession numbers: PX236525, PX236526, PX236527, PX236528 and PX236529).

Phenotypically, IR2.5 formed a fast‐growing, brownish‐white, round colony with a cottony texture and a slightly raised centre with aerated mycelium. The reverse was beige, with crescent‐shaped septate macroconidia, as evidenced by microscopic examination (Figure 10b). CR1.13 produced a circular colony with a white surface exhibiting dark beige pigmentation, velvety and cottony texture, and a raised centre with aerial mycelium. The reverse was light beige, slightly darker at the centre. Microscopically, the hyphae were septate and the spores elongated (Figure 10c). CR2.2 formed a circular cottony colony with raised centre, airy mycelium, dark beige and white in colour and a cream‐coloured reverse. Microscopy revealed septate hyphae and elongated, pointed spores (Figure 10d). MR2.8 developed a circular cottony colony with slightly irregular edges and a white centre, and pink to yellowish margins. The reverse was pale yellow and microscopic examination revealed spherical spores and *Purpureocillium*‐like conidiophores (Figure 10i).

Other isolates belonged to non‐*Fusarium* genera. Using molecular and phenotypic approaches, we identified isolates from genera *Cladosporium*, *Geotrichum* and *Didymella*. Phylogenetic analysis based on the ITS region showed that the CR1.3 and VA isolates clustered with the *Cladosporium* genus (99% identity), while the CD isolate clustered with *Geotrichum* sp. (100% identity; Figure S5). Phenotypically, CR1.3 formed a slightly oval, taupe‐grey colony with a white border, striations and woolly texture. The reverse was dark grey with a white rim. Microscopy revealed septate hyphae and slightly oval spores (Figure 10e). VA produced a raised, taupe‐grey, circular colony with a white rim and velvety texture and striations. The reverse was brownish grey with a beige border and striations (Figure 10f). CD developed a flat, circular, white colony with a creamy yeast‐like texture and a light beige reverse; microscopy revealed arthrospores (Figure 10g).

MC showed 100% identity with both *Didymella* and *Neoascochyta* (Figure S5). Its colony was circular with concentric rings in various shades of cream, a darker central region, a creamy texture and black and brown sand‐like granules. Microscopy revealed the presence of chlamydospores (Figure 10h).

Although the remaining isolates (VR1.10, VR2.8, CR1.4 and MR1.4) remained molecularly unidentified, we characterised them phenotypically. VR1.10 developed a circular creamy‐white colony with radial striations and a slightly woolly centre. The reverse was beige with stretch marks. Microscopy revealed septate hyphae (Figure 10j). VR2.8 formed a circular, raised, white colony with a cottony texture and concentric rings in various shades of pink. The reverse was light beige and microscopic examination revealed septate hyphae and spherical spores (Figure 10k). CR1.4 developed a circular, light‐yellow colony with a white border striation and a woolly texture. The reverse was beige. Microscopy revealed cenocytic hyphae and round spores (Figure 10l). MR1.4 formed a circular, white velvety colony with a raised cottony centre and creamy‐white reverse. Microscopy revealed the presence of chlamydospores (Figure 10m).

## 4 Discussion

In this study, we report the presence of PTEs in both soil and surface water from different sites along the urban Soto stream, located in the Hurlingham district, Argentina. Most detected concentrations were below the thresholds established by current Argentine regulations; however, several substances are either poorly regulated or lack national reference values altogether. For instance, Ba and Mn were present in the soil, which could indicate an uncontrolled discharge of these elements. Soil concentrations of Zn and Cr were comparable to those reported by a study performed in sediments from the Reconquista River near the Site V sampled for this research; however, Cr levels in our study were even lower (Tufo et al. 2021).

Across all sampling sites, B concentrations in soil exceeded the limit established for agricultural use (2 μg/g), despite the absence of specific thresholds for residential soils in the current Argentine regulatory framework (Decree 831/93, Law No. 24.051). Although Se was below the LQ, its actual concentrations may still surpass the residential soil limit of 3 μg/g established under the same regulation. If persistent and unmanaged, such accumulation could surpass acceptable thresholds over time, posing risks to biota and long‐term environmental health.

In surface water, Cd and Pb concentrations were below detection limits, but they may nonetheless exceed the regulatory thresholds for biota protection and direct recreational use (Decree 831/93, Law No. 24.051). For comparison, the Reconquista River Basin Committee (COMIREC) reported quantifiable levels of Cd in water samples from two sites on the Reconquista River in 2001—one in the middle basin and another in the lower basin—both surpassing the reference threshold for aquatic life protection (0.2 μg/L) (Decree 831/93, Law No. 24.051).

This study describes the presence of PTEs in both water and soil, as well as its concentration values. However, it is essential to acknowledge the methodological limitations inherent to the study when interpreting these results. Although spatial variations in PTEs concentrations were observed among sampling sites, only one composite soil sample was collected per site. However, the information obtained on the presence of PTEs at each site and time allowed for the characterisation of the soil from which the fungi were subsequently isolated. It is important to note that, although several PTEs were not detected, their presence cannot be ruled out. Several PTEs were below the limit of quantification, indicating that they may be present at low concentrations.

The present study represents a first assessment of PTEs in the urban Soto stream, based on samples collected during a single sampling campaign. Future studies including seasonal monitoring would allow a more comprehensive evaluation of temporal variations in PTE concentrations.

PTEs do not degrade naturally and tend to accumulate in water and soil (Briffa et al. 2020), which make their removal a major environmental challenge. Microorganisms represent a promising solution, as they can absorb, accumulate, reduce, oxidise or precipitate metals depending on their natural adsorption capacity (Kumar and Dwivedi 2021). With this in mind, the second objective of this study was to isolate PTEs‐tolerant fungal strains of Soto stream soil samples.

Preliminary qualitative screening revealed that 13 fungal strains grew in the presence of at least one of the tested PTEs. Radial mycelial growth was used as a preliminary indicator of probable tolerance to PTEs, allowing the identification of fungal isolates with potential for further evaluation in bioremediation studies. Among them, IR2.1 exhibited the broadest tolerance spectrum, with high growth in the presence of Mn, Zn (500 mg/L), Cu and Cr. Phylogenetic analysis identified IR2.1 as a member of the FIESC (Xia et al. 2019). The absence of discrimination between species within this complex is attributable to the fact that ITS sequences frequently fail to resolve the high similarity (98%–100%) and morphological homoplasy that exists among them (Wang et al. 2019). In this study, identification was based on characteristic morphological features, EF1‐α sequencing, and differential responses to PTEs (Hami et al. 2021). Notably, plant‐microorganism interactions can enhance soil remediation capacity, and *Fusarium incarnatum* combined with perennial ryegrass has shown the highest remediation rate (49.35%) after 45 days, with a significantly lower soil Zn concentration than the control group (Zhang et al. 2022).

Three additional isolates—IR2.5 (*F. solani‐*like), CR1.13 (*F. keratoplasticum‐*like) and CR2.2 (*F. witzenhausense‐*like)—belonged to the FSSC, as defined by Cherhi et al. (2015). Morphologically, all exhibited septate mycelia but differed in colony features: IR2.5 was fast growing with a cottony texture, and crescent‐shaped macroconidia; CR1.13 formed a velvety colony with elongated spores; and CR2.2 developed a beige‐white colony with long, pointed spores. Tolerance profiles were also distinct: IR2.5 tolerated Mn, Zn, Cu and Pb, which aligned with previous reports of the high Zn and Cu biosorption potential of *Fusarium Solani* (El Sayed and El‐Sayed 2020; Liaquat et al. 2021). CR1.13 (*Fusarium keratoplasticum*‐like) tolerated Mn, Cu and Pb, but was sensitive to high concentrations of Zn, while CR2.2 (*F. witzenhausense*‐like) showed moderate tolerance to Mn and Pb. All three showed Pb moderate tolerance (200 mg/L), consistent with findings that inactivated *Fusarium* strains (ZSY and MJY) can effectively remove Pb (II) from aqueous environments (Long et al. 2019).

The isolate MR2.8 was identified as *Purpureocillium lavendulum‐like* strain showing tolerance to high levels of Zn, Cu (500 mg/L), Cr (100 mg/L) and Pb (500 mg/L), and moderate growth under Cd exposure. This agrees with the detected high CdCl_2_ tolerance reported for the nematophagous fungus *P. lavendulum* YMF1.683 (Li et al. 2024). To date, no available report indicates tolerance of this species to the other metals evaluated in this study. Although MR2.8 shared sequence identity with *P. lalicium*, its phenotype and metal tolerance profile supported classification as a member of *P. lavendulum*. Another unidentified isolate, VR2.8, showed remarkable Cd tolerance and exhibited morphology consistent with *Paecilomyces lilacinus*, a species previously reported to efficiently biosorb Cd (Zeng et al. 2010) and to mitigate the damage caused by PTE stress in *Solanum lycopersicum* plants (Musa et al. 2023).

Other isolates included *Cladosporium* sp. (VA) and *Geotrichum* sp. (CD), both of which showed moderate Mn tolerance, in agreement with Mn biosorption capacity reported for *Cladosporium halotolerans* (Mota et al. 2020). *Cladosporium* sp. also exhibit good Cd(II) uptake potential (Roșca et al. 2024), warranting further evaluation of the VA isolate. The MC isolate, showing molecular and phenotypic similarities to *Didymella* and *Neoascochyta* genera, responded to Pb exposure in a manner comparable to *Fusarium* spp., although PTEs resistance has not been reported for these two genera.

Taken together, these findings suggest that PTEs tolerance is both species‐ and strain‐dependent, involving mechanisms such as biosorption, vacuolar sequestration, efflux transporters and oxidative stress regulation (Tucker et al. 2004; Liu et al. 2020; Ragasa et al. 2021). While PTEs such as Zn and Cu are essential micronutrients at low concentrations, others like Pb and Cd are strictly toxic, impairing fungal growth through oxidative damage and metabolic inhibition (Savi et al. 2013; Robinson et al. 2021). Among all isolates, *F. equiseti*‐like IR2.1 and *Purpureocillium lavendulum*‐like MR2.8 consistently exhibited broad tolerance to multiple metals, underscoring their potential as promising candidates for mycoremediation strategies in contaminated environments.

From a taxonomic standpoint, some fungal isolates could not be fully resolved using ITS and TEF markers alone, suggesting that additional loci or genomic approaches may be required for precise identification at the species level.

Future research should incorporate multi‐season sampling to more accurately capture the temporal variability of PTEs concentrations and the responses of fungal communities. Functional bioremediation assays in liquid cultures and controlled microcosms are necessary to validate PTE removal under realistic conditions. Further studies should address key PTEs attenuation mechanisms, such as biosorption, bioaccumulation and transformation, and explore fungal‐plant and fungal‐bacterial interactions. Finally, testing fungal‐based materials (e.g., biochar composites or immobilised biomass) in real wastewater or mining effluents is essential for assessing their practical applicability.

In summary, this study provides baseline information on PTEs presence in an environment that has been impacted by human activity, in terms of water and soil. In addition, the findings of this study indicate that *Fusarium equiseti* and *Purpureocillium lavendulum* could be promising candidates due to their high PTEs tolerance indices. This suggests that they have strong potential for their future application in mycoremediation strategies applicable to wastewater, industrial effluents and mining‐contaminated environments. While the study offers important first insights into the fungal community associated with a contaminated ecosystem, further research is required to assess the functional capacity of these isolates for PTEs removal under controlled and field conditions.

## Author Contributions

**Ludmila López Arias:** investigation, writing – original draft, writing – review and editing, methodology, formal analysis, validation, conceptualization, funding acquisition, project administration. **Juan José Herrera:** methodology, investigation. **Daiana Betsabé Monzón Bechara Roucos:** methodology, investigation. **Nadia Stefania Alvarez:** methodology, investigation. **Melisa Leone:** conceptualization, investigation, funding acquisition, writing – original draft, writing – review and editing, methodology, project administration, supervision, data curation, formal analysis, validation, visualization.

## Funding

This work was supported by the Universidad Nacional de Hurlingham (UNAHUR) (Grants PIUNAHUR 2022 and PIUNAHUR 2024).

## Conflicts of Interest

The authors declare no conflicts of interest.

## Supporting information


**Table S1:** Number of fungal isolates from each sampling site (I, M, V, C) showing tolerance to specific PTEs at the indicated concentrations. The number of tolerant isolates per PTE is shown for Cd, Cr, Cu, Pb, Zn and Mn. Tolerance was assessed by growth on PGA plates supplemented with the respective element.
**Table S2:** Tolerance index (TI) values of selected fungal isolates exposed to different concentrations of PTEs (a) Mn, (b) Zn, (c) Cu, (d) Pb, (e) Cr and (f) Cd. Growth was assessed on PGA plates supplemented with the corresponding PTE at two concentrations. TI was calculated as the ratio between radial growth on metal‐supplemented medium and growth on control medium (PGA without PTE).
**Figure S1:**. Mycelial growth kinetics of MC in the presence of different PTEs at two concentrations. (a) Mn, (b) Cu and (c) Pb. Line graphs the progression of mycelial area (cm^2^) over time on PGA control medium (C) and PGA supplemented with the corresponding PTE. Asterisks (*) indicate significant differences compared with the control (*p* < 0.05). Representative images illustrate colony morphology under control and metal stress conditions. Scale bars (upper left corner) represent 1 cm.
**Figure S2:** Mycelial growth kinetics of MR1.4 in the presence of different PTEs at two concentrations. (a) Mn, (b) Zn and (c) Pb. Line graphs show the progression of mycelial area (cm^2^) over time on PGA control medium (C) and PGA supplemented with the corresponding PTE. Representative images illustrate colony morphology under control and metal stress conditions. Scale bars (upper left corner) represent 1 cm.
**Figure S3:** Mycelial growth kinetics of VR2.8 in the presence of different PTEs at two concentrations. (a) Mn, (b) Zn, (c) Cu and (d) Cd. Line graphs show the progression of mycelial area (cm^2^) over time on PGA control medium (C) and PGA supplemented with the corresponding PTE. Asterisks (*/**) indicate significant differences compared with the control (*p* < 0.05). Representative images illustrate colony morphology under control and metal stress conditions. Scale bars (upper left corner) represent 1 cm.
**Figure S4:** Mycelial growth kinetics of CR1.4 in the presence of different PTEs at two concentrations. (a) Mn, (b) Zn and (c) Cr. Line graphs show the progression of mycelial area (cm^2^) over time on PGA control medium (C) and PGA supplemented with the corresponding PTE. Asterisks (*/**) indicate significant differences compared with the control (*p* < 0.05). Representative images illustrate colony morphology under control and metal stress conditions. Scale bars (upper left corner) represent 1 cm.
**Figure S5:** Phylogenetic tree based on the ITS fragment. The evolutionary history was inferred using the maximum likelihood method and the Tamura‐Nei model. The percentage of trees in which the associated taxa clustered together is shown next to the branches. This analysis involved 10 nucleotide sequences. There were a total of 312 positions in the final dataset.

## Data Availability

The data generated and analysed during this study are included in this published article and its Supporting Information. The nucleotide sequences obtained in this study have been deposited in the GenBank database under accession numbers PX236525–PX236529. Other data that support the findings of this study are available from the corresponding author upon reasonable request.
