# Supplementary material for: Detection of Potentially Toxic Elements and Tolerant Native Fungi Presence in an Urban Stream in Argentina
Source: Environ Microbiol Rep. 2026 May 21;18(3):e70350. doi: 10.1111/1758-2229.70350 (PMC13240332; doi:10.1111/1758-2229.70350)
Supplement: Supplementary file 1 — Table S1: Number of fungal isolates from each sampling site (I, M, V, C) showing tolerance to specific PTEs at the indicated concentrations. The number of tolerant isolates per PTE is shown for Cd, Cr, Cu, Pb, Zn and Mn. Tolerance was assessed by growth on PGA plates supplemented with the respective element. Table S2: Tolerance index (TI) values of selected fungal isolates exposed to different concentrations of PTEs (a) Mn, (b) Zn, (c) Cu, (d) Pb, (e) Cr and (f) Cd. Growth was assessed on PGA plates supplemented with the corresponding PTE at two concentrations. TI was calculated as the ratio between radial growth on metal‐supplemented medium and growth on control medium (PGA without PTE). Figure S1:. Mycelial growth kinetics of MC in the presence of different PTEs at two concentrations. (a) Mn, (b) Cu and (c) Pb. Line graphs the progression of mycelial area (cm2) over time on PGA control medium (C) and PGA supplemented with the corresponding PTE. Asterisks (*) indicate significant differences compared with the control (p < 0.05). Representative images illustrate colony morphology under control and metal stress conditions. Scale bars (upper left corner) represent 1 cm. Figure S2: Mycelial growth kinetics of MR1.4 in the presence of different PTEs at two concentrations. (a) Mn, (b) Zn and (c) Pb. Line graphs show the progression of mycelial area (cm2) over time on PGA control medium (C) and PGA supplemented with the corresponding PTE. Representative images illustrate colony morphology under control and metal stress conditions. Scale bars (upper left corner) represent 1 cm. Figure S3: Mycelial growth kinetics of VR2.8 in the presence of different PTEs at two concentrations. (a) Mn, (b) Zn, (c) Cu and (d) Cd. Line graphs show the progression of mycelial area (cm2) over time on PGA control medium (C) and PGA supplemented with the corresponding PTE. Asterisks (*/**) indicate significant differences compared with the control (p < 0.05). Representative images illustrate col [file EMI4-18-e70350-s001.docx]

Table S1. Number of fungal isolates from each sampling site (I, M, V, C) showing tolerance to specific PTEs at the indicated concentrations. The number of tolerant isolates per PTE is shown for Cd, Cr, Cu, Pb, Zn and Mn. Tolerance was assessed by growth on PGA plates supplemented with the respective element.

| **Site** | **Total Isolates** | **PTEs concentration** | **Number of tolerant isolates** |
| --- | --- | --- | --- |
| I | 5 | **Cd (50 mg/L)** | 0 |
|  |  | **Cr (50 mg/L)** | 2 |
|  |  | **Cu (200 mg/L)** | 2 |
|  |  | **Pb (200 mg/L)** | 1 |
|  |  | **Zn (200 mg/L)** | 2 |
|  |  | **Mn (200 mg/L)** | 2 |
| M | 6 | **Cd (50 mg/L)** | 1 |
|  |  | **Cr (50 mg/L)** | 1 |
|  |  | **Cu (200 mg/L)** | 2 |
|  |  | **Pb (200 mg/L)** | 3 |
|  |  | **Zn (200 mg/L)** | 2 |
|  |  | **Mn (200 mg/L)** | 3 |
| V | 8 | **Cd (50 mg/L)** | 1 |
|  |  | **Cr (50 mg/L)** | 0 |
|  |  | **Cu (200 mg/L)** | 2 |
|  |  | **Pb (200 mg/L)** | 0 |
|  |  | **Zn (200 mg/L)** | 1 |
|  |  | **Mn (200 mg/L)** | 3 |
| C | 5 | **Cd (50 mg/L)** | 1 |
|  |  | **Cr (50 mg/L)** | 3 |
|  |  | **Cu (200 mg/L)** | 2 |
|  |  | **Pb (200 mg/L)** | 2 |
|  |  | **Zn (200 mg/L)** | 5 |
|  |  | **Mn (200 mg/L)** | 5 |

Table S2. Tolerance index (TI) values of selected fungal isolates exposed to different concentrations of PTEs a) Mn, b) Zn, c) Cu, d) Pb, e) Cr and f) Cd. Growth was assessed on PGA plates supplemented with the corresponding PTE at two concentrations. TI was calculated as the ratio between radial growth on metal-supplemented medium and growth on control medium (PGA without PTE).

| **a)** |  |  | **b)** |  |  | **c)** |  |  |
| --- | --- | --- | --- | --- | --- | --- | --- | --- |
|  | **Mn** | |  | **Zn** | |  | **Cu** | |
|  | 200 mg/L | 500 mg/L |  | 200 mg/L | 500 mg/L |  | 200 mg/L | 500 mg/L |
| **IR2.1** | 2.04 | 2.05 | **IR2.1** | 2.00 | 2.00 | **IR2.1** | 1.94 | 1.73 |
| **IR2.5** | 1.00 | 0.99 | **IR2.5** | 0.98 | 0.96 | **IR2.5** | 0.99 | 1.00 |
| **MC** | 1.05 | 0.99 | **MR1.4** | 0.93 | 0.85 | **MC** | 0.96 | 0.94 |
| **MR1.4** | 0.96 | 0.97 | **MR2.8** | 5.38 | 3.63 | **MR2.8** | 4.10 | 3.86 |
| **MR2.8** | 0.80 | 0.75 | **VR2.8** | 0.89 | 0.84 | **VR1.10** | 0.89 | 0.25 |
| **VA** | 1.05 | 1.02 | **CD** | 1.02 | 0.41 | **VR2.8** | 1.00 | 0.84 |
| **VR1.10** | 0.89 | 0.79 | **CR1.3** | 0.82 | 0.30 | **CD** | 0.99 | 0.43 |
| **VR2.8** | 0.84 | 0.88 | **CR1.4** | 1.57 | 0.68 | **CR2.2** | 1.04 | 1.02 |
| **CD** | 0.97 | 0.94 | **CR1.13** | 0.92 | 0.87 |  |  |  |
| **CR1.3** | 0.99 | 0.82 | **CR2.2** | 0.99 | 0.96 |  |  |  |
| **CR1.4** | 0.90 | 0.77 |  |  |  |  |  |  |
| **CR1.13** | 1.00 | 1.00 |  |  |  |  |  |  |
| **CR2.2** | 1.00 | 1.00 |  |  |  |  |  |  |
|  |  |  |  |  |  |  |  |  |
| **d)** |  |  | **e)** |  |  | **f)** |  |  |
|  | **Pb** | |  | **Cr** | |  | **Cd** | |
|  | 200 mg/L | 500 mg/L |  | 50 mg/L | 100 mg/L |  | 50 mg/L | 100 mg/L |
| **IR2.5** | 0.90 | 0.10 | **IR2.1** | 1.50 | 1.44 | **MR2.8** | 0.89 | 0.50 |
| **MC** | 0.98 | 0.48 | **IR2.5** | 1.00 | 1.00 | **VR2.8** | 1.03 | 0.98 |
| **MR1.4** | 1.00 | 1.12 | **MR2.8** | 2.98 | 2.10 | **CR1.13** | 0.99 | 0.29 |
| **MR2.8** | 0.47 | 1.54 | **CR1.4** | 0.91 | 0.52 |  |  |  |
| **CR1.13** | 1.00 | 0.14 | **CR1.13** | 0.95 | 0.19 |  |  |  |
| **CR2.2** | 1.00 | 0.17 | **CR2.2** | 1.00 | 0.85 |  |  |  |


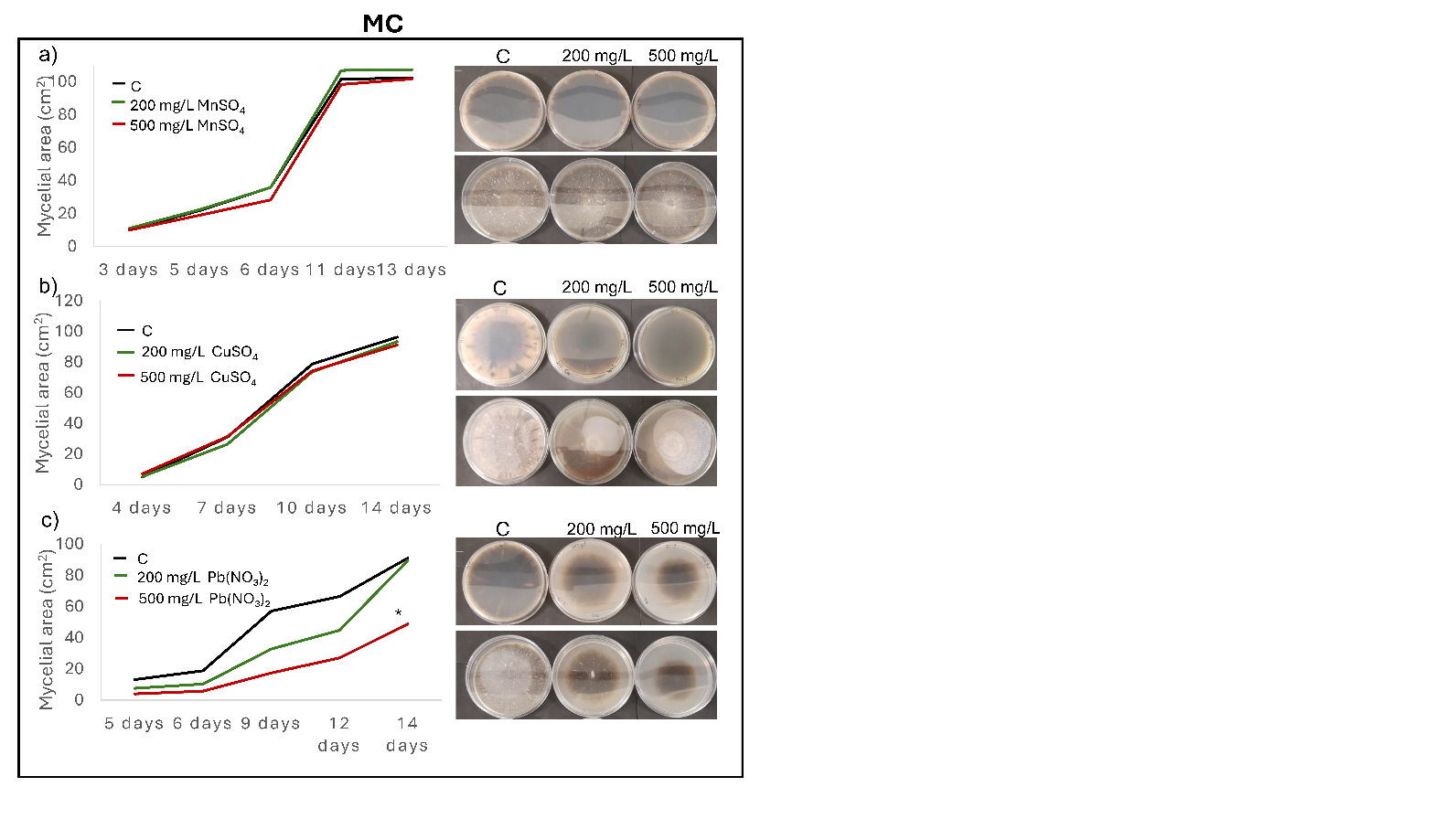


Figure S1. Mycelial growth kinetics of MC in the presence of different PTEs at two concentrations. a) Mn, b) Cu and c) Pb. Line graphs the progression of mycelial area (cm²) over time on PGA control medium (C) and PGA supplemented with the corresponding PTE Asterisks (*) indicate significant differences compared with the control (p<0.05). Representative images illustrate colony morphology under control and metal stress conditions. Scale bars (upper left corner) represent 1 cm.


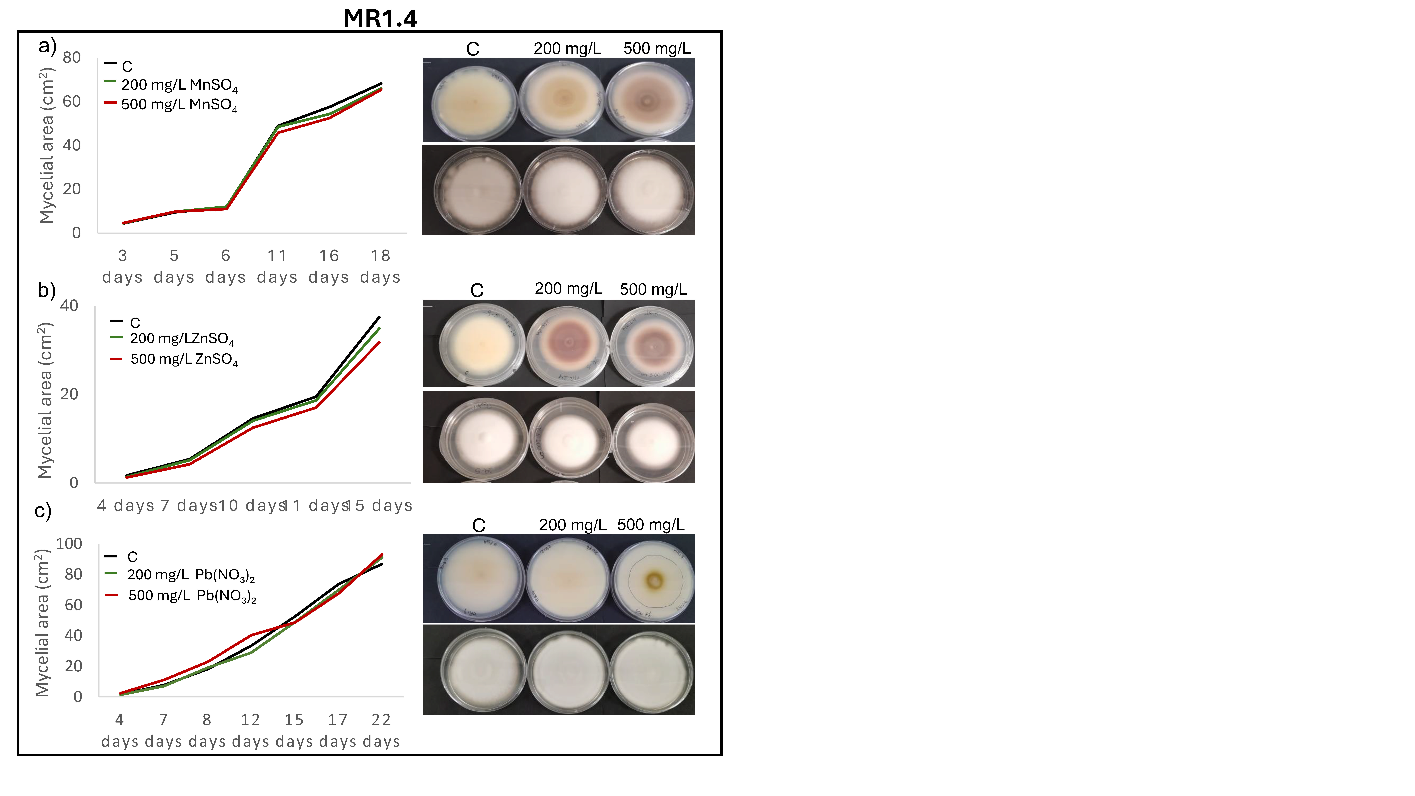


Figure S2. Mycelial growth kinetics of MR1.4 in the presence of different PTEs at two concentrations. a) Mn, b) Zn and c) Pb. Line graphs show the progression of mycelial area (cm²) over time on PGA control medium (C) and PGA supplemented with the corresponding PTE. Representative images illustrate colony morphology under control and metal stress conditions. Scale bars (upper left corner) represent 1 cm.


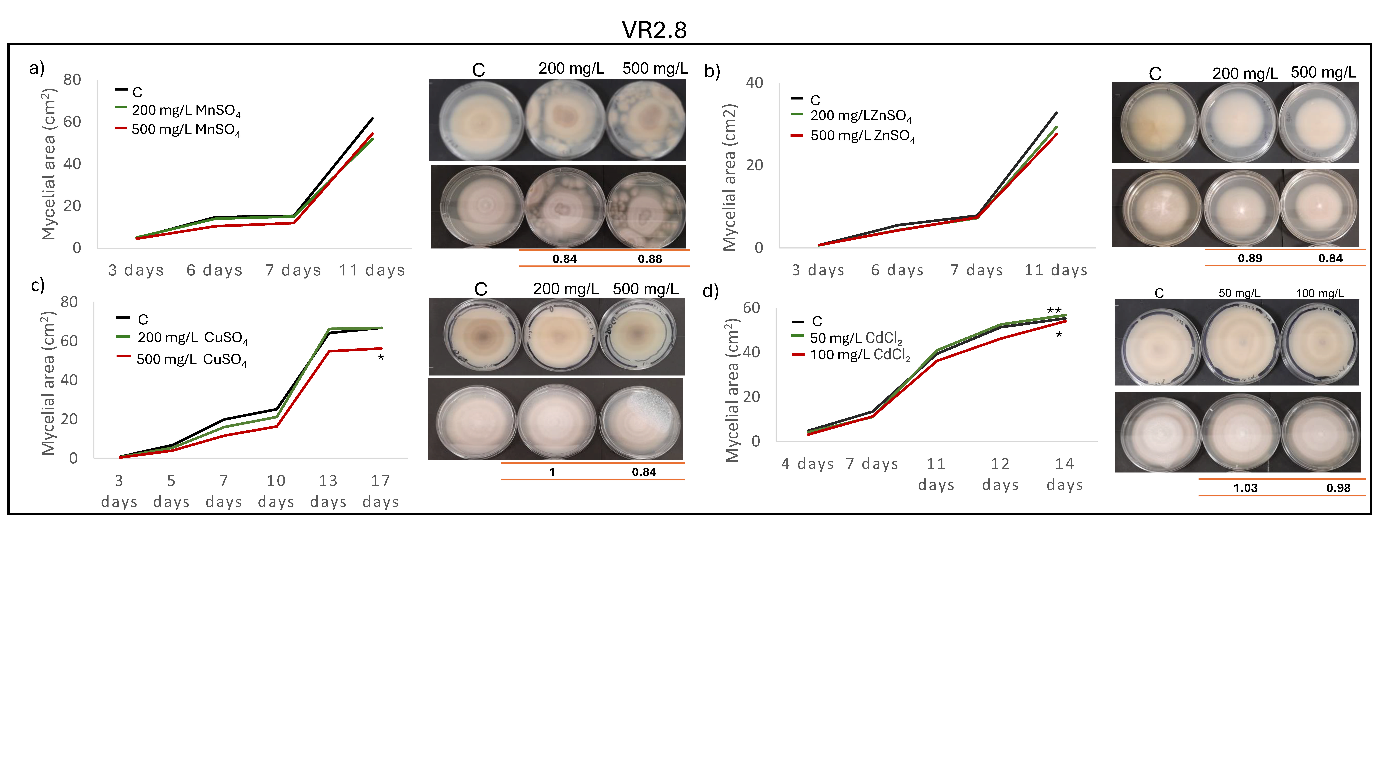


Figure S3. Mycelial growth kinetics of VR2.8 in the presence of different PTEs at two concentrations. a) Mn, b) Zn, c) Cu and d) Cd. Line graphs show the progression of mycelial area (cm²) over time on PGA control medium (C) and PGA supplemented with the corresponding PTE. Asterisks (*/**) indicate significant differences compared with the control (p<0.05). Representative images illustrate colony morphology under control and metal stress conditions. Scale bars (upper left corner) represent 1 cm.


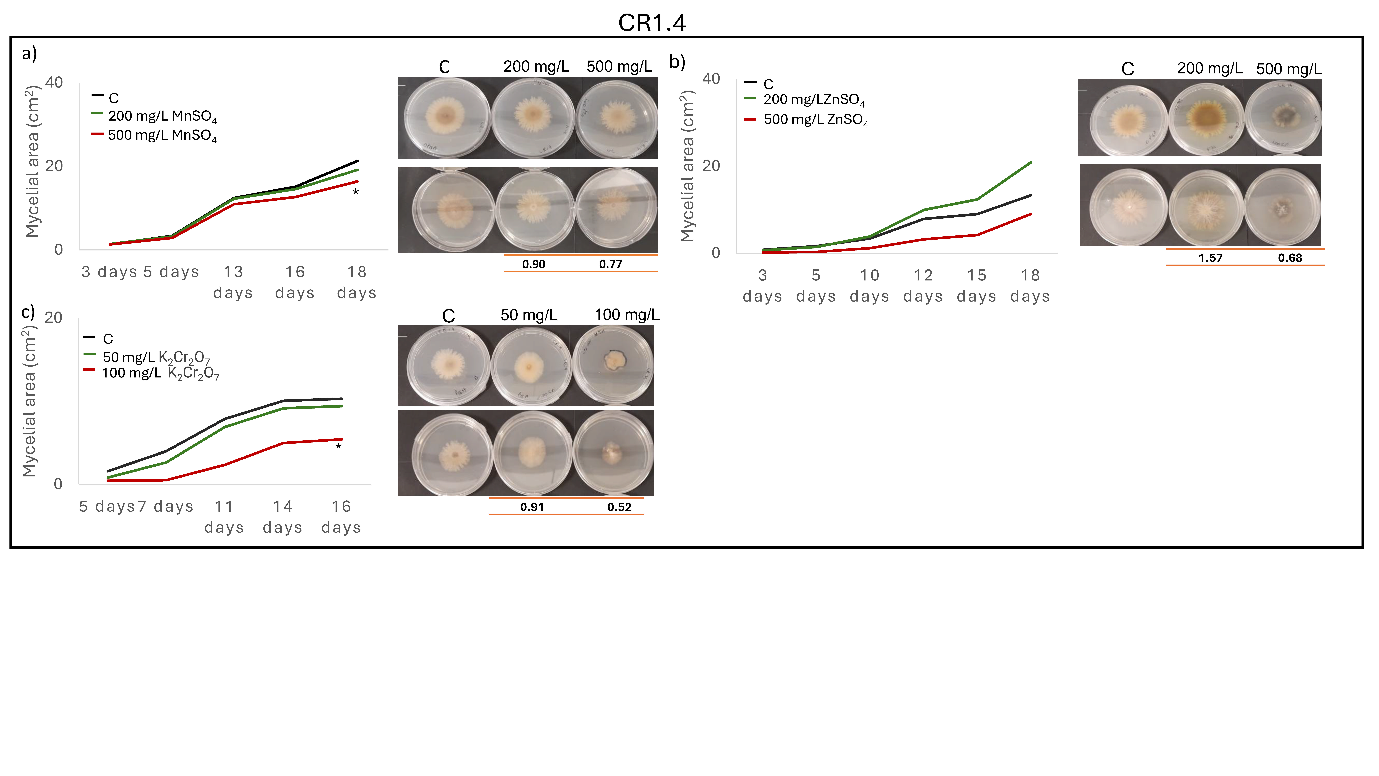


Figure S4. Mycelial growth kinetics of CR1.4 in the presence of different PTEs at two concentrations. a) Mn, b) Zn and c) Cr. Line graphs show the progression of mycelial area (cm²) over time on PGA control medium (C) and PGA supplemented with the corresponding PTE. Asterisks (*/**) indicate significant differences compared with the control (p<0.05). Representative images illustrate colony morphology under control and metal stress conditions. Scale bars (upper left corner) represent 1 cm.


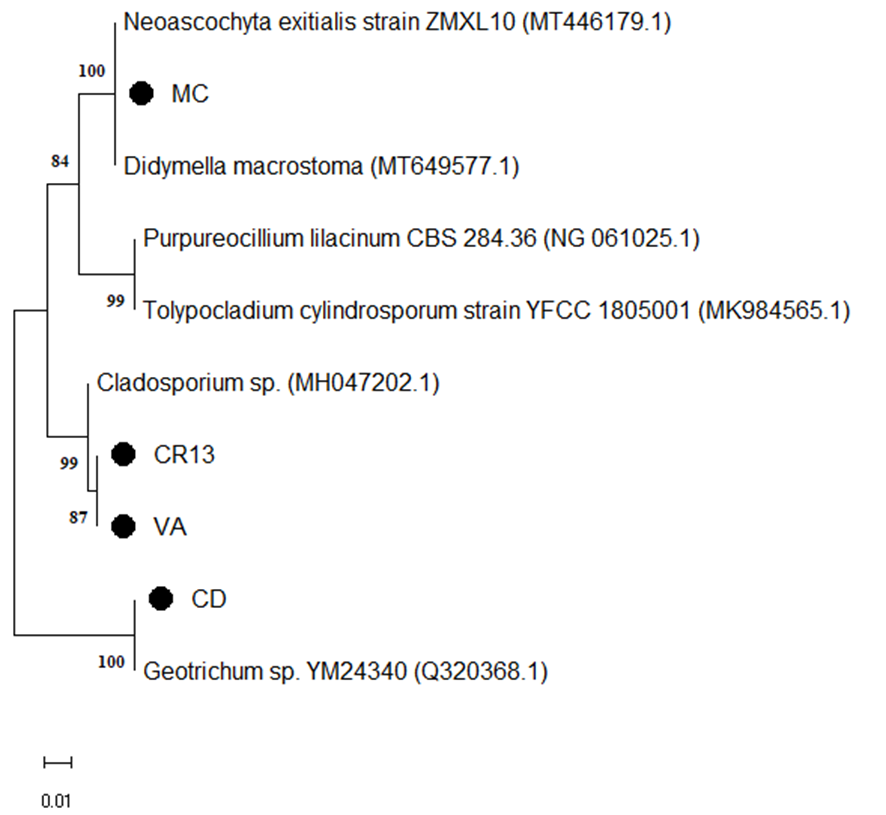


Figure S5. Phylogenetic tree based on the ITS fragment. The evolutionary history was inferred using the Maximum Likelihood method and the Tamura-Nei model. The percentage of trees in which the associated taxa clustered together is shown next to the branches. This analysis involved 10 nucleotide sequences. There were a total of 312 positions in the final dataset.
